# *Tu-Teng-Cao* Extract Alleviates Monosodium Urate-Induced Acute Gouty Arthritis in Rats by Inhibiting Uric Acid and Inflammation

**DOI:** 10.1155/2020/3095624

**Published:** 2020-04-21

**Authors:** Rongmei Yao, Zihan Geng, Xin Mao, Yanyan Bao, Shanshan Guo, Lei Bao, Jing Sun, Yingjie Gao, Yingli Xu, Bo Guo, Fengxian Meng, Xiaolan Cui

**Affiliations:** ^1^Institute of Chinese Materia Medica, China Academy of Chinese Medical Sciences, Beijing 100700, China; ^2^College of Traditional Chinese Medicine, North China University of Sciences and Technology, Tangshan 063210, China; ^3^Department of Critical Care Medicine, Henan Provincial People's Hospital, Zhengzhou 450003, China; ^4^Dongfang Hospital Affiliated to Beijing University of Chinese Medicine, Beijing 100700, China

## Abstract

Gouty arthritis is an inflammatory joint disease closely related to hyperuricemia. It is characterized by deposition of monosodium urate crystals in the joints, resulting in an intense inflammatory process and pain. Control of hyperuricemia and anti-inflammation treatments are the main therapeutic approaches. However, the commonly used drugs for inhibiting uric acid and acute gouty arthritis have obvious gastrointestinal and renal toxicity; thus, there is an urgency to develop new alternative therapeutic drugs. An extract of *Tu-Teng-Cao* (TTC), a compound drug used in traditional Chinese medicine, has been widely applied to the clinical treatment of arthritis. In this study, we investigated the therapeutic effects of TTC on gouty arthritis. In this study, an animal model of acute gouty arthritis with hyperuricemia was established using potassium oxonate and monosodium urate crystals. After treatment with TTC, the results showed obvious therapeutic effects on the rat model of acute gouty arthritis. The treatment significantly attenuated the degree of ankle swelling, inflammation, and dysfunction index, and the levels of proinflammatory cytokines. In addition, TTC has significant antihyperuricemia activity in rats with hyperuricemia induced by potassium oxonate. Histological evaluation showed that TTC relieved pathological damage in rats with acute gouty arthritis induced by monosodium urate crystals. All the groups treated with TTC showed improvement in cartilage degeneration, cell degeneration, synovial hyperplasia, and inflammatory cell invasion in the ankle joint of rats. TTC significantly alleviated swelling, inflammation, and bleeding of the renal corpuscle and convoluted tubules of rats. The results of this study suggest that TTC is capable of treating gouty arthritis and decreasing ankle injury through the control of uric acid and inflammation.

## 1. Introduction

Gout is a metabolic disease characterized by hyperuricemia and deposition of urate crystals into the joints and accompanied by inflammatory reactions [1]. Acute arthritis is the most common initial symptom of primary gout. The patient presents with redness, swelling, and severe pain in the affected joints and surrounding tissues [2]. Epidemiological evidence suggests that this disease is more common in men aged >40 years and postmenopausal women [3]. The prevalence of gouty arthritis has been increased in recent decades, as a result of the increase in intake of high-fat and purine-rich foods, as well as genetic factors [4–6]. Notably, the incidence of gouty arthritis in younger individuals is gradually increasing, especially in young males [7].

In the pathogenesis of acute gouty arthritis, monosodium urate (MSU) crystals are formed due to supersaturation of uric acid concentration in the joint. This effect triggers the body's innate immune response, leading to an induction of inflammation cascades, causing severe inflammation in the joints [8]. In this process, MSU is recognized by the resident macrophages of tissue, thereby initiating an inflammatory response and recruiting neutrophils. During this process, a large number of inflammatory factors and cellular contents are released, which is considered to be a key to the pathogenesis of acute gouty arthritis [9]. MSU crystals deposited in the tissue result in activation of the NLRP3 inflammasome [10, 11]. Activation of the NLRP3 inflammasome signaling pathway leads to the release of the proinflammatory cytokine interleukin-1*β* (IL-1*β*), which in turn induces the expression of a number of proinflammatory factors, such as tumour necrosis factor-alpha (TNF-*α*), IL-6, and IL-8. These cytokines can recruit monocytes and neutrophils flowing into the synovial membrane of the joint, causing local inflammatory reactions (e.g., increased vascular permeability, plasma exudation, fever, and swelling) and leading to severe joint pain, swelling, and dysfunction. This is the main pathological feature of gouty arthritis [12, 13]. Excessive and uninterrupted inflammation can cause damage to healthy tissue, ultimately leading to cartilage degeneration and joint damage [14].

Control of hyperuricemia and treatment that reduces inflammation are the major therapeutic approaches against gouty arthritis. Relevant drugs are mainly classified into uric acid-lowering drugs and acute-risk anti-inflammatory drugs [15]. The former group of drugs can effectively reduce the content of uric acid in the serum and are further divided into drugs that inhibit its production and promote its excretion [16, 17]. The drugs that inhibit the production of uric acid are represented by allopurinol, which acts by inhibiting the enzyme xanthine oxidase. The drugs that promote uric acid excretion are represented by benzbromarone, which acts by inhibiting the reabsorption of uric acid in the kidneys. However, these two types of drugs are associated with strong toxicity to the liver and kidneys and can easily lead to cardiovascular abnormalities [18]. More importantly, the currently available uric acid-lowering drugs do not exert an effect on the inflammatory pain of gout. Thus, anti-inflammatory treatment has become the first choice against acute episodes of gout. At present, commonly used drugs mainly include colchicine, nonsteroidal anti-inflammatory drugs, and glucocorticoids. Although these drugs are effective, adverse reactions (e.g., renal toxicity or gastrointestinal bleeding) are also prominent [19]. New biopharmaceuticals can be used for the treatment of gouty arthritis; however, the high cost and inherent drawbacks of biopharmaceuticals limit the application of these drugs [20]. The currently available first-line drugs for the treatment of gouty arthritis have been linked to several adverse effects. Thus, it is particularly urgent and important to identify alternative therapeutic strategies.

Traditional Chinese medicine has been using herbal drugs to treat gout for >2,000 years. Recently, studies have shown that numerous herbal drugs and their active ingredients can be used against MSU crystal-induced gouty arthritis, with multiple targets and low toxicity [21–23]. TTC is a crude extract from the traditional Chinese medicine compound *Tu-Teng-Cao*, which has been applied to the clinical treatment of acute-onset gout arthritis [24]. This study used the potassium oxonate- and MSU-induced acute gout arthritis rat model to evaluate the efficacy of TTC in treating hyperuricemia and gouty inflammatory arthritis, confirm the anti-inflammatory and analgesic effects, and preliminarily discuss the potential anti-inflammatory mechanisms of TTC. The aim of this investigation was to shed light on the future application of TTC as a drug for gouty arthritis offering easier storage and oral administration than traditional Chinese medicine compound decoctions.

## 2. Materials and Methods

### 2.1. Preparation Process and Quality Standard of TTC

#### 2.1.1. Plant Materials

The main recipe of traditional Chinese medicine compound TTC was *Polygonum cuspidatum Sieb.et Zucc.*, *Sargentodoxa cuneate (Oliv.) Rehd. et Wils., Smilax glabra Roxb.*, *Lonicera japonica Thunb.*, *Lysimachia christinae Hance., Phellodendron chinense Schneid., Commiphora myrrha Engl.,* and *Angelica dahurica (Fisch. ex Hoffm.) Benth. et Hook. f.* All herbal drugs were purchased from Anguo Herb Market (China). The material was identified by Dr. Xirong He and complied with the specification of Pharmacopoeia of the People's Republic of China (2015). A voucher specimen of the material is retained in the Centre of Chinese Medicine Preparations, Institute of Chinese Materia Medica, China Academy of Chinese Medical Sciences (Beijing, China).

#### 2.1.2. Preparation of TTC

The herbal drugs were crushed using a blender, and distilled water was added in a liquid-solid ratio of 1 : 10 (g/mL). The herbs were extracted twice at 95°C for 1.5 h each time (R-501 rotary evaporator; Gaoke Instrument Factory, Gongyi, China). The extract solution was centrifuged, and the supernatant was concentrated in vacuum. The concentrated solution was precipitated with 95% (v/v) ethanol and incubated overnight. The precipitates were collected through centrifugation and dried by spray to obtain TTC [25].

#### 2.1.3. Establishment of Quality Standards

The main pharmacological ingredients of this traditional Chinese medicine compound are *Lonicera japonica Thunb.* and *Polygonum cuspidatum Sieb. et Zucc.* The main active ingredients in *Lonicera japonica Thunb.* are chlorogenic acid and loganin. The main active ingredients in *Polygonum cuspidatum Sieb. et Zucc*. are polydatin and emodin. Chlorogenic acid and emodin are not specific components of *Lonicera japonica Thunb.* and *Polygonum cuspidatum Sieb. et Zucc.* Therefore, this preparation selects loganin and polydatin as the content measurement targets for the evaluation of quality. The content of the product was determined using high-performance liquid chromatography (e2695; Waters Corp., Massachusetts, USA), the content of loganin was 0.6 mg/g, and the content of polydatin was 1.0 mg/g [25].

### 2.2. Chemicals and Reagents

Potassium oxonate was purchased from Shanghai Yuanye Bio-Technology Co., Ltd. (Shanghai, China). Uric acid sodium salt was obtained from Sigma-Aldrich (St. Louis, MO, USA). The uric acid assay kit was purchased from Nanjing Jiancheng Bioengineering Institute (Nanjing, China). Enzyme-linked immunosorbent assay (ELISA) kits for TNF-*α*, IL-1*β*, and IL-6 were obtained from Cloud-Clone Corp. (Wuhan, China). All other reagents used were standard laboratory reagents of analytical grade and were purchased locally.

### 2.3. Preparation of MSU Crystals

MSU crystals were prepared by crystallizing the supersaturated solution of uric acid with some modifications as previously described [3]. Briefly, 5 g uric acid was dissolved and heated in 1,000 ml H_2_O with sodium hydroxide (9 mL/0.5 N) and adjusted to pH 8.9 at 60°C. The solution was gradually cooled at room temperature and stored overnight at 4°C to allow crystallization. The crystals were sterilized by heating at 100°C for 2 h. The resulting MSU crystals (needle-shaped, length: 5–20 *μ*m) were suspended in endotoxin-free phosphate-buffered saline (PBS) (25 mg/mL). The bacterial endotoxin contamination in MSU crystals was assessed using limulus amebocyte lysate (<0.01 EU/10 mg) and detection procedures in accordance with the Chinese Pharmacopoeia [26].

### 2.4. Animals

Sprague Dawley rats (male, specific pathogen free, weight: 230–270 g) were purchased from Vital River Laboratory Animal Technology Co., Ltd. (Beijing, China). All animals were maintained in a standard laboratory conditioned at a temperature of 25 ± 2°C with 50–55% relative humidity and a 12 h light/dark cycle. All the rats were fed with standard food and pure water. Animal experimentation and the corresponding protocol (no. 2018-0042) were approved by the Animal Ethics Committee of the Institute of Chinese Materia Medica China Academy of Chinese Medical Sciences. All the procedures were in strict accordance with the People's Republic of China legislation regarding the use and care of laboratory animals.

### 2.5. Drug Dosage

The adult conventional dosage of TTC was 0.1 g/kg/day. The high (1 g/kg/day), medium (0.5 g/kg/day), and low (0.25 g/kg/day) doses used in this study were converted based on the adult dose, which was equivalent to twice, equal to, and half the human dose, respectively. The standard drug used in this study was colchicine (0.3 mg/kg/day), which was equivalent to the adult dose [27].

### 2.6. Animal Model of Acute Gouty Arthritis with Hyperuricemia in Rats and Experimental Design

After a 7-day acclimatization period, an experimental animal model of hyperuricemia was induced using potassium oxonate (uricase inhibitor). Briefly, potassium oxonate (1.5 g/kg/day) dissolved in distilled water was orally administered to 70 rats; the dosing volume was 1 mL/100 g body weight, once daily for 21 consecutive days. In addition, 10 rats were used to form the normal control group (administration of distilled water only). Blood samples were collected from the eyelids of model rats at weeks 1, 2, and 3, and the levels of uric acid in the serum were measured. Rats with blood uric acid levels >110 *μ*mol/L at week 3 indicated that the model was successfully induced. Animals with high or low levels of uric acid in the blood were removed, and 50 rats were selected for the subsequent experiment.

**Figure 1:**
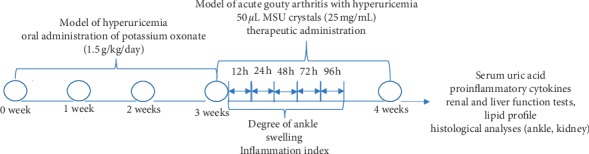
Experimental design. A hyperuricemia model was established during the first 3 weeks through the daily administration of potassium oxonate. Subsequently, a model of acute gouty arthritis with hyperuricemia was established via injection of MSU crystals, as described in the Materials and Methods section. This was followed by the administration of TTC, colchicine, or PBS (control) once daily for 7 days. The degree of ankle swelling, inflammation index, and dysfunction index were assessed at 12, 24, 48, 72, and 96 h after the injection of MSU crystals. Rats were sacrificed to test for proinflammatory cytokines, renal and liver function, lipid profile, and histological analyses.

The successfully established hyperuricemia model rats were randomly divided into six groups (*n* = 10 per group): model control, normal control, colchicine, and TTC high dose (TTC-H), TTC medium dose (TTC-M), and TTC low dose (TTC-L). All rats were anesthetized with 2.5% isoflurane, followed by injection of 50 *μ*L MSU crystals (25 mg/mL) or normal saline into the medial side of the right tibiotarsal joint (ankle) of each rat to further establish the model of acute gouty arthritis with hyperuricemia. Of note, the contralateral bulging of the joint capsule was the standard for drug injection [28]. After the administration of MSU, each group intragastrically received TTC or colchicine once daily for 7 days. The normal control group and the model control group were treated with PBS (Figure 1).

### 2.7. Assessment of Inflammation

#### 2.7.1. Degree of Ankle Swelling

Following the injection of MSU crystals, the width of the right ankle joint at different time intervals was measured using a vernier scale. The left and right diameters (*a*) and the anteroposterior diameter (*b*) of the ankle joint were measured before establishing the arthritis model, and 12, 24, 48, 72, and 96 h after the administration of MSU crystals [29]. The ankle joint volume was calculated using the following formula: ankle joint volume = 1/2 × *ab*^2^.

#### 2.7.2. Inflammation Index and Dysfunction Index

The progression of acute arthritis was evaluated by macroscopic scoring of the ankle joint. Data were recorded prior to establishing the arthritis model, and 12, 24, 48, 72, and 96 h after the administration of MSU crystals. The inflammation and dysfunction scores of the rats were visually determined by two independent observers.

The following criteria were used to score inflammation [30]:

  Grade 0 (0 points): ankle joint is normal without any inflammatory reaction
  Grade 1 (2 points): joints have erythema of skin, mild swelling, and visible bony marks
  Grade 2 (4 points): joints are obviously red and swollen, bony landmarks disappear, and swelling is limited to the joints
  Grade 3 (6 points): swelling outside the joint, degree of inflammatory reaction is more severe, ability of the foot is weakened, and the foot is often lifted off the ground

The following criteria were used to score dysfunction [31]:

  Grade 0 (0 points): normal gait, and both feet are evenly grounded
  Grade 1 (2 points): toes are not unfolded, and foot is slightly limp
  Grade 2 (4 points): foot is bent and clearly limping, and toes are on the ground
  Grade 3 (6 points): foot completely lifted off the ground, three-legged gait

#### 2.7.3. Proinflammatory Cytokines

After the end of the experimental period (day 8), the animals were sacrificed through euthanasia. The articular cavities were irrigated with 0.5 mL normal saline, the joint fluid was collected, and the samples were centrifuged at 3,000 rpm/min for 15 min. The levels of TNF-*α*, IL-1*β*, and IL-6 were measured using ELISA kits, according to the instructions provided by the manufacturer.

### 2.8. Determination of Uric Acid

On day 8 after treatment, blood samples were collected from the eye sockets of rats. The samples were maintained for 30 min at room temperature and subsequently centrifuged at 3,000 rpm/min for 15 min. The concentration of uric acid in the serum was determined through the phosphotungstic acid method [32] using a test kit, according to the instructions provided by the manufacturer.

### 2.9. Determination of Renal Function

The renal function (i.e., blood urea and creatinine) before and after administration was detected using a biochemical analyser (TBA-40FR; Toshiba Corp., Tokyo, Japan). The serum samples were the same as described in Section 2.8.

### 2.10. Histological Analysis

The ankle joints and kidneys of rats were fixed in 4% paraformaldehyde, and the ankles were decalcified using 10% ethylenediaminetetraacetic acid. Tissues were dehydrated by processing in different levels of alcohol, mixed with chloroform, immersed in paraffin, and cut into 5 *μ*m thick slices. The tissue slices were stained with haematoxylin and eosin dye to show the extent of the sliding film and kidney damage. Images were independently evaluated using a light microscope (BX53; Olympus Corp., Tokyo, Japan) in a blinded manner.

Ankle histology scores were as follows (−∼+++) [6]:
    “−”: normal
    “+”: mild infiltration of mononuclear cells
    “++”: moderate inflammatory cell immersion and cartilage destruction and mild oedema of intertissue
    “+++”: massive inflammatory cells into the synovial and joint space and significant oedema of intertissue and cartilage cells
Kidney histology scores were as follows (−∼+++) [33]:
    “−”: normal
    “+”: minor infiltration of mononuclear cells
    “++”: mild swelling of glomerular and renal tubes and slight bleeding
    “+++”: significant swelling of glomerular and renal tubes and mild bleeding

### 2.11. Statistical Analysis

Measurement data were expressed as mean ± standard deviation and analysed using the GraphPad Prism 6.0 software. One-way analysis of variance was used, followed by Student's Newman–Keul's test. Histological results, as well as inflammation and dysfunction indices, were evaluated using the Statistical Package for the Social Sciences (SPSS) version 17.0 software (SPSS Inc., Chicago, IL, USA), using the rank-sum test. ^*∗*^*P* < 0.05 denoted statistical significance.

## 3. Results

### 3.1. TTC Ameliorated Inflammation in Rats

#### 3.1.1. TTC Mitigated the Degree of Ankle Swelling

**Figure 2:**
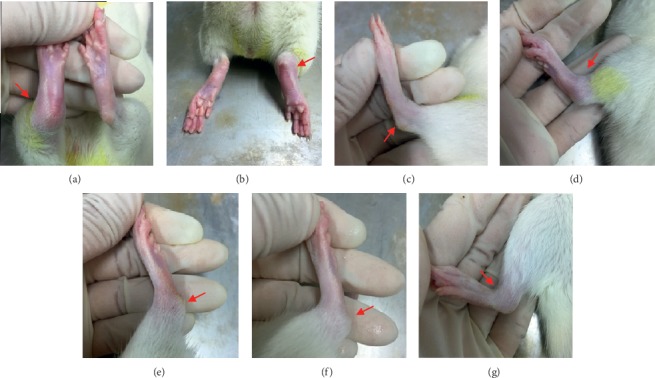
MSU crystals induced gouty arthritis in Sprague Dawley rats. These figures show the macroscopic signs observed in each group 24 h after the injection of MSU or PBS into the ankle joints. (a, b) Model control group (injection of MSU crystals). (c) Normal control group (injection of PBS). (d) Colchicine group. (e–g) TTC-H, TTC-M, and TTC-L (1, 0.5, and 0.25 g/kg, respectively) groups.

MSU crystals were synthesized with uric acid and sodium hydroxide and diluted in endotoxin-free PBS prior to injection into the right ankle joint of rats. At 24 h after the injection of MSU crystals, significant redness, swelling, and deformity were observed at the ankle joints of the rats. In the model group, the ankle joint and toes were significantly red and swollen, bony landmarks disappeared, and the degree of inflammation was severe (Figures 2(a) and 2(b)). There was no inflammatory reaction in the normal group, and bony landmarks were obvious (Figure 2(c)). The ankle joints in the TTC treatment groups were swollen; however, the swelling was confined to the joints without involving the toes, and the degree of inflammation was significantly lower than that noted in the model group (Figures 2(e)–2(g)). The severity of the inflammatory response was evaluated by the volume of ankle joint swelling. The volume of ankle joints was tested at 12, 24, 48, 72, and 96 h after the injection of MSU crystals. As shown in Table 1, there was no difference in the volume of ankle joints prior to establishing the model. The volume of the ankle joints in the model groups increased significantly after the injection of MSU crystals, indicating that the model was successfully established. The volume of the ankle joint at each time point was significantly mitigated after the administration of TTC, especially in the TTC-M group at 24, 48, and 72 h after administration. Notably, the volume in the TTC-L group at 24 h was significantly reduced compared with that reported in the model control group.

#### 3.1.2. TTC Reduced the Inflammation and Dysfunction Indices

After the injection of MSU crystals into the ankle joint, the rats progressively developed a series of arthritis symptoms. In the preliminary experiment, we observed the acute phase of inflammation within 96 h after the administration of MSU. Hence, we measured the following index and cytokine levels at 0–96 h. The occurrence of arthritis was obvious after 12 h. The joints of rats in the model group gradually swelled and could not move freely, and the inflammation index scores were significantly higher than those recorded for the normal control group. The inflammatory scores of the three doses of TTC granules decreased after 24, 48, and 72 h. At 48 h after the administration of TTC, the TTC-M group showed a significant therapeutic effect, which was statistically different from that noted in the model group (Table 2). Inflammation can cause pain and dysfunction; thus, we performed behavioural testing of rats to evaluate the degree of inflammation. After the injection of MSU crystals, the index of joint dysfunction in the model group was significantly higher than that determined for the normal control group. At 24, 48, 72, and 96 h, the dysfunction scores of the three TTC groups were not statistically significant compared with those calculated for the model control group (Table 3).

#### 3.1.3. TTC Decreased the Level of Proinflammatory Cytokines in the Joint Fluid

**Figure 3:**
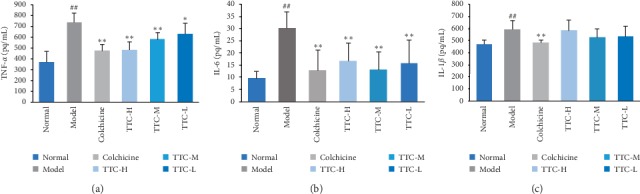
Effects of TTC on proinflammatory cytokines in MSU crystal-induced rats. The samples were ankle lavage fluid of rats. The evaluation time was 7 days after the administration of TTC. Data represent the mean ± standard deviation of values obtained from six animals. ^##^*P* < 0.01 compared with the normal control group; ^*∗*^*P* < 0.05 and ^*∗∗*^*P* < 0.01 compared with the hyperuricemic control group (ANOVA followed by Student's Newman–Keul's test).

Acute gouty arthritis is characterized by the high expression of proinflammatory cytokines. The levels of proinflammatory cytokines (TNF-*α*, IL-1*β*, and IL-6) were measured in the joint fluid of MSU crystal-induced rats using ELISA to ascertain whether TTC inhibited this characteristic. Treatment with TTC showed significantly lower levels of TNF-*α* and IL-6 in the MSU crystal-induced rats versus the model group. The level of IL-1*β* was not significantly different compared with that reported in the model control group (Figure 3). These results indicate that TTC can alleviate the symptoms of acute arthritis induced by MSU.

### 3.2. TTC Ameliorated Hyperuricemia in Rats

**Figure 4:**
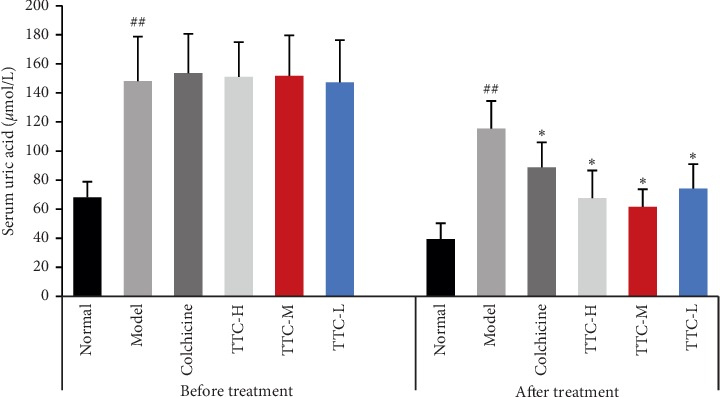
Effects of TTC on serum uric acid in hyperuricemic rats. Data represent the mean ± standard deviation of data obtained from 10 animals. ^##^*P* < 0.01 compared with the normal control group; ^*∗*^*P* < 0.05 compared with the hyperuricemic control group (ANOVA followed by Student's Newman–Keul's test).

We evaluated the level of serum uric acid in the groups of treated rats to confirm whether TTC can also ameliorate hyperuricemia. Administration with potassium oxonate for 3 weeks caused hyperuricemia in rats, and the levels of uric acid in the serum were significantly increased compared with those measured in the normal control group. As expected, the increased level of uric acid was inhibited by treatment with TTC. A 7-day treatment with TTC (1, 0.5, and 0.25 g/kg) significantly reduced the levels of serum uric acid compared with those observed for the hyperuricemic control group (Figure 4).

### 3.3. TTC Improved the Renal Function of Rats Treated with MSU Crystals

The results of the renal function test showed after the injection of MSU crystals, the serum levels of blood urea and creatinine in the model group were significantly higher than those measured in the normal control group. After 7 days of administration, there was no significant difference in all TTC treatment groups compared with the model control group (Table 4).

### 3.4. TTC Improved Ankle Joint and Kidney Lesions in Rats Treated with MSU Crystals

Histopathological analysis was performed by evaluating the lesions of ankle joints (i.e., periosteum and cartilage) and kidneys (i.e., renal corpuscle and convoluted tubule) in rats treated with MSU crystals to assess whether TTC could improve the histological lesions in rats.

**Figure 5:**
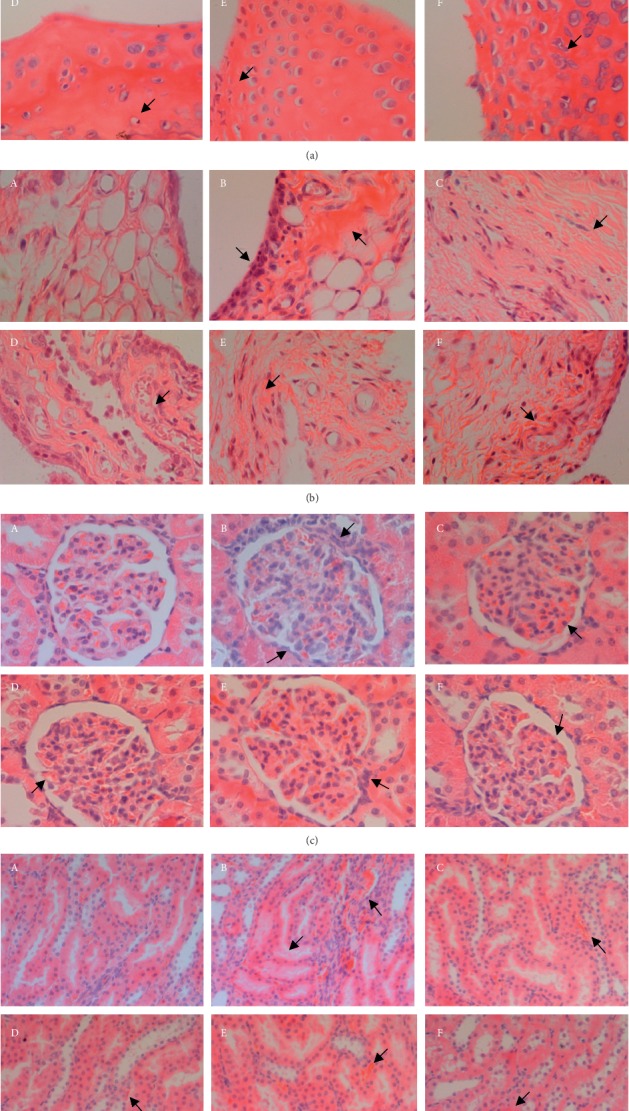
Effect of TTC on MSU crystal-induced rat ankle joints and kidney lesions (*n* = 10). (A) Normal control group (injection of PBS). (B) Model control group (injection of MSU crystals). (C) Colchicine group. (D–F) TTC-H, TTC-M, and TTC-L (1, 0.5, and 0.25 g/kg, respectively) groups. (a) Cartilage: arrows indicating chondrocyte degeneration, and the nucleus disappears (B–C); enlargement of chondrocytes, and the nucleus becomes smaller or lysed (E–F). (b) Periosteum: arrows indicating thickening of synovia, myxedema, and increased number of cells (B–F). (c) Renal corpuscles: arrows indicating hypertrophy of glomerular mesangial cells, congestion in the glomeruli, and segmental increase in the number of parietal cells (B–F). (d) Convoluted tubules: arrows indicating swelling of renal tubules, shedding of endometrial cells, interstitial inflammation, stasis, and other lesions (B–F). Arrows indicating typical lesions; scale bar = 100 *μ*m; original magnification ×400.

In the normal group, the cartilage and periosteum of the ankle joints in rats showed no degeneration and the structure was normal (Figures 5(a)A and 5(b)A). Obvious pathological changes were observed in the ankle joint of the rats in the model control group, showing chondrocyte vacuole degeneration, significant synovial thickening, increased inflammatory cells, swelling of interstitial tissue, and exudate (Figures 5(a)B and 5(b)B). All TTC treatment groups exhibited improved or alleviated cartilage degeneration, cell degeneration, synovial hyperplasia, and inflammatory cell invasion in the ankle joint of rats (Figures 5(a)D–F and 5(b)D–F). The results in Table 5 show that there was no lesion in the ankle joints of the normal control group. In addition, the pathological score of the model control group was greatly increased, which was significantly different from that reported in the normal group. After 7 days of TTC administration, the pathological scores of ankle joints in all TTC groups were not significantly different compared with those calculated in the model control group (Table 5).

In the normal group, the kidney structure of the rats was normal without obvious lesions (Figure 5(c)A). In the model control group, the renal corpuscle of rats was swollen, with blood stasis and increased mesangial cells. Simultaneously, the kidney convoluted tubules were swollen, the intimal cells were detached, and the interstitial tubules were inflamed with blood stasis (Figures 5(c)B and 5(d)B). The pathological damage in the three TTC groups was significantly lower than that noted in the model control group. In the normal control group, the kidneys of rats did not have visible lesions (Figures 5(c)D–F and 5(d)D–F). The results in Table 6 show that there was no lesion in the normal control group, and the pathological scores of renal corpuscles and convoluted tubules in the model control group were significantly increased compared with those recorded in the normal group. After 7 days of TTC administration, the pathological score was significantly reduced. The evaluation of convoluted tubules showed that there were significant differences between the three TTC groups and the model control group. The evaluation of renal corpuscles showed that there were significant differences between the TTC-H (1 g/kg) group and the model control group (Table 6).

## 4. Discussion

Gouty arthritis is an inflammatory response triggered by the abnormal metabolism of uric acid. It is characterized by intense pain, redness, and swelling around the joints and connective tissues. The management of acute flares of gout is extremely difficult for clinicians. Currently, gouty arthritis is typically treated with nonsteroidal anti-inflammatory drugs, colchicine, adrenocortical hormones, or biological agents. However, these drugs are linked to serious side effects and lack of safety in patients with comorbidities [34, 35]. Existing evidence suggests that traditional Chinese medicines, as one of the most popular drugs of complementary and alternative medicine, possess a low toxicity profile and exhibit beneficial efficacy in the treatment of gouty arthritis [22–24, 36]. In this study, the effects of an extract (TTC) derived from a novel herbal formula on gouty arthritis were evaluated in rats injected with MSU crystals.

Potassium oxonate is a uric acid enzyme inhibitor, which can cause hyperuricemia in animals through intragastric or intraperitoneal injection. This model has been widely used in the screening of uric acid-lowering drugs [37, 38]. Recent studies indicate that MSU crystals are closely related to the pathology of gouty arthritis and are among the most effective proinflammatory stimuli [39]. The formation of MSU crystals into the joint cavity caused acute inflammation, demonstrating equivalent symptoms to those found in clinical gout. This model has good predictive value for the clinical efficacy against gout arthritis at the research level [28]. Based on previous methods, we have made improvements and attempted to construct a new animal model. We successfully established a rat model of hyperuricemia with acute gouty arthritis through intragastric administration of potassium oxonate for 3 weeks and injection of MSU crystals into the ankle joint.

The phagocytosis of MSU crystals by macrophages activated the formation of the inflammasome. Subsequently, it induced high expression of a variety of proinflammatory cytokines, which are directly responsible for the influx of neutrophils and monocytes into the synovium, resulting in the enhancement of the inflammatory response [40]. Arthritis causes pain, and movement of the affected joint is typically reduced, which ultimately affects the ability to perform activities. Twenty-four hours after injection, significant swelling of the ankle joint was observed, accompanied by joint dysfunction. Of note, after the administration of TTC, the ankle swelling in rats was obviously decreased. The inflammatory and dysfunction indices were also lower than those determined in the model group. The continuous observations in this experiment showed that the ankle joint volume at 24, 48, and 72 h and the inflammation index at 48 h in the TTC-M group were statistically different from those calculated in the model group. The TTC-L group showed a statistically significant difference in ankle joint volume at 24 h versus the model group. The TTC-H group did not show a significant therapeutic effect in terms of reducing the ankle joint volume, inflammation and dysfunction indices, and pathological damage. Combining the above results, we could observe that there was a dose-effect relationship between the TTC-M and TTC-L groups. Such observations are often noted in pharmacological experiments of traditional Chinese medicine. The reasons may be related to the complex dose-effect relationship of traditional Chinese medicine compound preparations. The TTC-M group represents the dose routinely used in the clinical setting. In the experiment, the concentration of the drug in the TTC-H group is twice the recommended dosage. The preparation of Chinese medicine compounds is complicated, and the excessive concentration limits the absorption of the active ingredients by the body, so pharmacodynamics effect is often not ideal.

Furthermore, MSU crystal-stimulated neutrophils release oxygen radicals, proteolytic enzymes, and proinflammatory cytokines, leading to damage of the articulation [41]. After the administration of TTC with three doses for 1 week, histopathological assessment revealed a treatment effect. TTC reduced synovial hyperplasia, cartilage damage, and bone erosion. Histopathological examination showed that treatment with TTC in all three groups alleviated pathological damage in the ankle joints of rats. However, the difference versus the model group was not statistically significant. The results were attributed to the rapid self-healing ability of rats [42]. The rat ankle joint is sensitive to inflammatory response and commonly used for drug development against arthritis. Nevertheless, the recovery ability of in rats with inflammatory injury was also strong. The continuous observations in this experiment showed that the model group of rats without any drug intervention exhibited the most severe ankle swelling at 48 and 72 h after the administration of MSU crystals into the ankle joint, which was gradually reduced after 96 h. By the end of the experiment (i.e., 7 days), most of the rats did not show obvious redness and swelling.

MSU crystals are one of the most effective proinflammatory stimuli, which can trigger, amplify, and sustain a strong inflammatory reaction in the joint cavity [3]. A previous study demonstrated that MSU crystals stimulate the synthesis and release of IL-1*β* [43]. IL-1*β* is the pivotal inflammatory mediator that regulates the differentiation, proliferation, and apoptosis of cells in gouty arthritis. In addition, IL-1*β* can induce the expression of a wide range of cytokines and chemokines, such as TNF-*α*, IL-6, IL-17, and monocyte chemoattractant protein-1. This leads to a large influx of neutrophils into the synovium, which is the main pathological feature of gout arthritis. Furthermore, MSU crystals can also stimulate the secretion of TNF-*α* and IL-6 by macrophages, neutrophils, and monocyte-macrophages, which induce acute inflammation. These persistent inflammatory reactions cause subsequent tissue damage [44]. Therefore, blocking the secretion of inflammatory mediators from MSU crystal-activated macrophages is beneficial to the control and management of acute gout arthritis. In this study, our results indicated that TTC significantly reduced the secretion of cytokines TNF-*α* and IL-6 in the synovial fluid of rats treated with MSU crystals. Moreover, the secretion of IL-1*β* tended to decrease compared with that reported in the model group, although there was no statistically significant difference.

Hyperuricemia is a disease in which the level of uric acid in the blood is elevated due to a decrease in purine metabolism or excretion of uric acid from the body. In the present study, a hyperuricemic rat model was generated using potassium oxonate to investigate the pharmacological effect of TTC on the reduction of uric acid function. After 3 weeks of intragastric administration of potassium oxonate, the levels of serum uric acid were significantly higher than those observed in normal rats (reached ≥110 *μ*mol/L), which was in accordance with the expected model requirements. Potassium oxonate is an inhibitor of the uric acid enzyme and has demonstrated strong activity both *in vivo* and *in vitro*. It can effectively inhibit the activity of uricase and increase the levels of uric acid in the experimental animals [45]. In the present study, the levels of uric acid in the serum were measured before and after the administration of TTC. TTC significantly downregulated the increased levels of serum uric acid. These results suggested that TTC plays a role in the amelioration of hyperuricemia.

Uric acid is closely related to kidney disease. It can be handled by the kidneys and the process is complicated, requiring glomerular filtration, reabsorption of filtered urate, tubular secretion, and finally secretion and reabsorption. In the presence of abnormal metabolism of uric acid, the kidney is the most likely organ to be damaged. The deposition of uric acid crystals in the renal corpuscle and tubulointerstitium may cause an inflammatory reaction along with the release of cytokines. In this situation, intrarenal precipitation of uric acid also contributes to renal insufficiency [46]. In the present study, renal function in each group was measured after the administration of TTC. The analysis found that TTC exerted a protective effect on the kidneys. At the end of the experiment, pathological examination of the kidneys showed that TTC significantly alleviated pathological symptoms, such as swelling, cell degeneration, inflammation, and haemorrhage of renal corpuscles and convoluted tubules in rats.

## 5. Conclusions

We established an optimized rat model of acute gouty arthritis with hyperuricemia through administration of potassium oxonate and MSU crystals and clarified the overall effects of TTC against gouty arthritis. The results showed that TTC could inhibit the inflammatory cascade and regulate the metabolism of uric acid. Therefore, this extract may be an effective drug for the treatment of hyperuricemia and gouty arthritis.

## Figures and Tables

**Table 1:** Effects of TTC on the volume of ankle joints in rats with acute gouty arthritis.

Group	Dose	Unit	Before injection of MSU	After injection of MSU				
				12 h	24 h	48 h	72 h	96 h
Normal	—	mm^3^	323.95 ± 23.48	324.18 ± 23.70	324.02 ± 23.39	325.07 ± 23.46	325.64 ± 23.50	327.02 ± 23.03
Model	—	mm^3^	317.59 ± 22.49	707.45 ± 102.95^##^	922.78 ± 121.11^##^	746.59 ± 130.50^##^	489.88 ± 79.71^##^	428.44 ± 74.84^##^
Colchicine	0.3 mg/kg	mm^3^	320.42 ± 23.83	635.78 ± 126.16	788.64 ± 119.54^*∗*^	564.06 ± 66.87^*∗*^	420.93 ± 54.30^*∗*^	376.24 ± 45.33
TTC-H	1 g/kg	mm^3^	318.56 ± 28.88	702.25 ± 89.91	864.86 ± 91.61	665.73 ± 88.41	446.62 ± 72.97	395.95 ± 74.66
TTC-M	0.5 g/kg	mm^3^	314.06 ± 24.93	646.97 ± 102.36	809.73 ± 141.09^*∗*^	625.73 ± 116.46^*∗*^	426.27 ± 79.66^*∗*^	385.67 ± 70.19
TTC-L	0.25 g/kg	mm^3^	319.05 ± 26.65	671.16 ± 122.82	817.61 ± 150.29^*∗*^	653.92 ± 164.91	504.37 ± 91.02	424.15 ± 83.42

Measurement data represent the mean ± standard deviation of 10 animals. One-way ANOVA followed by Student's Newman–Keul's test was used for statistical analysis. ^##^*P* < 0.01 compared with the normal control group; ^*∗*^*P* < 0.05 compared with the model control group.

**Table 2:** Effects of TTC on the inflammation index in rats with acute gouty arthritis.

Group	Unit	After injection of MSU									
		12 h					24 h				
		Grade 0	Grade 1	Grade 2	Grade 3	*P* value	Grade 0	Grade 1	Grade 2	Grade 3	*P* value

Normal	pcs	10	0	0	0		10	0	0	0	
Model	pcs	0	4	5	1	^##^	0	0	4	6	^##^
Colchicine	pcs	0	6	3	1		0	4	5	1	^*∗*^
TTC-H	pcs	0	4	5	1		0	2	6	2	
TTC-M	pcs	0	5	4	1		0	3	5	2	
TTC-L	pcs	0	4	5	1		0	1	7	2	

Group	Unit	48 h					72 h				
		Grade 0	Grade 1	Grade 2	Grade 3	*P* value	Grade 0	Grade 1	Grade 2	Grade 3	*P* value

Normal	pcs	10	0	0	0		10	0	0	0	
Model	pcs	0	0	3	7	^##^	0	6	2	2	^##^
Colchicine	pcs	0	4	6	0	^*∗∗*^	4	5	1	0	^*∗*^
TTC-H	pcs	0	0	7	3		2	5	2	1	
TTC-M	pcs	0	3	5	2	^*∗*^	2	6	2	0	
TTC-L	pcs	0	1	6	3		2	4	4	0	

Group	Unit	96 h									
		Grade 0	Grade 1	Grade 2	Grade 3	*P* value					

Normal	pcs	10	0	0	0						
Model	pcs	3	5	2	0	^##^					
Colchicine	pcs	6	4	0	0						
TTC-H	pcs	4	4	2	0						
TTC-M	pcs	5	5	0	0						
TTC-L	pcs	3	6	1	0						

**Table 3:** Effects of TTC on the dysfunction index in rats with acute gouty arthritis.

Group	Unit	After injection of MSU									
		12 h					24 h				
		Grade 0	Grade 1	Grade 2	Grade 3	*P* value	Grade 0	Grade 1	Grade 2	Grade 3	*P* value

Normal	pcs	10	0	0	0		10	0	0	0	
Model	pcs	0	7	3	0	^##^	0	3	3	4	^##^
Colchicine	pcs	0	8	2	0		0	6	3	1	
TTC-H	pcs	0	8	2	0		0	4	4	2	
TTC-M	pcs	0	8	2	0		0	5	3	2	
TTC-L	pcs	0	8	2	0		0	4	4	2	

Group	Unit	48 h					72 h				
		Grade 0	Grade 1	Grade 2	Grade 3	*P* value	Grade 0	Grade 1	Grade 2	Grade 3	*P* value

Normal	pcs	10	0	0	0		10	0	0	0	
Model	pcs	0	3	3	4	^##^	1	5	2	2	^##^
Colchicine	pcs	0	5	2	3		4	5	1	0	
TTC-H	pcs	0	4	3	3		1	6	2	1	
TTC-M	pcs	0	5	2	3		3	6	1	0	
TTC-L	pcs	0	3	4	3		2	4	4	0	

Group	Unit	96 h									
		Grade 0	Grade 1	Grade 2	Grade 3	*P* value					

Normal	pcs	10	0	0	0						
Model	pcs	3	6	1	0	^##^					
Colchicine	pcs	6	4	0	0						
TTC-H	pcs	4	5	1	0						
TTC-M	pcs	5	5	0	0						
TTC-L	pcs	3	6	1	0						

Measurement data represent the interquartile range of 10 animals. The rank-sum test for multiple sets of independent samples was used for statistical analysis. ^##^*P* < 0.01 compared with the normal control group; ^*∗*^*P* < 0.05 and ^*∗∗*^*P* < 0.01 compared with the model control group.

**Table 4:** Effects of TTC on the renal function of rats with acute gouty arthritis.

Group	Dose	Blood urea (mmol/L)	Creatinine (*μ*mol/L)
Normal	—	6.42 ± 0.73	24.67 ± 4.36
Model	—	7.79 ± 0.94^##^	28.60 ± 0.74^#^
Colchicine	0.3 mg	7.32 ± 1.21	27.93 ± 5.13
TTC-H	1 g/kg	7.42 ± 1.89	27.63 ± 4.64
TTC-M	0.5 g/kg	7.41 ± 1.53	28.90 ± 7.87
TTC-L	0.25 g/kg	7.40 ± 1.01	26.36 ± 2.83

Measurement data represent the mean ± standard deviation of data obtained from 10 animals. One-way ANOVA and Student's Newman–Keul's test were used for statistical analysis. ^#^*P* < 0.05 and ^##^*P* < 0.01 compared with the normal control group; ^*∗*^*P* < 0.05 compared with the model control group.

**Table 5:** Effects of TTC on ankle joint lesions in rats with acute gouty arthritis.

Group	Unit	Tissue	Grade of lesions				*P* value
			−	+	++	+++	
Normal	pcs	Periosteum	10				
		Cartilage	10				

Model	pcs	Periosteum			9	1	^##^
		Cartilage			7	3	^##^

Colchicine	pcs	Periosteum		2	6	2	
		Cartilage		3	6	1	

TTC-H	pcs	Periosteum		1	9		
		Cartilage		2	6	2	

TTC-M	pcs	Periosteum		2	8		
		Cartilage		1	9		

TTC-L	pcs	Periosteum		5	4	1	
		Cartilage		2	8		

**Table 6:** Effects of TTC on kidney lesions in rats with acute gouty arthritis.

Group	Unit	Tissue	Grade of lesions				*P* value
			−	+	++	+++	
Normal	pcs	Renal corpuscles	10				
		Convoluted tubules	10				

Model	pcs	Renal corpuscles			8	2	^##^
		Convoluted tubules			4	6	^##^

Colchicine	pcs	Renal corpuscles		1	9		
		Convoluted tubules		2	8		^*∗∗*^

TTC-H	pcs	Renal corpuscles		5	5		^*∗*^
		Convoluted tubules		1	8	1	^*∗∗*^

TTC-M	pcs	Renal corpuscles		2	8		
		Convoluted tubules		5	5		^*∗∗*^

TTC-L	pcs	Renal corpuscles		2	8		
		Convoluted tubules		3	7		^*∗∗*^

Measurement data represent the interquartile range obtained from 10 animals. The rank-sum test for multiple sets of independent samples was used for statistical analysis. ^##^*P* < 0.01 compared with the normal control group; ^*∗*^*P* < 0.05 and ^*∗∗*^*P* < 0.01 compared with the model control group.

## Data Availability

The data used to support the findings of this study are available from the corresponding author upon request.
